# Mind to Move: A Narrative Review of Individualized Running Biomechanics Beyond the Spring-Mass Model

**DOI:** 10.1007/s40279-026-02449-w

**Published:** 2026-05-12

**Authors:** Cyrille Gindre, Thibault Lussiana, Bastiaan Breine, Aurélien Patoz

**Affiliations:** 1 Research and Development Department, Volodalen, 1023 Crissier, Switzerland; 2 Research and Development Department, Volodalen, 39270 Chavéria, France; 3 Université Marie et Louis Pasteur, SINERGIES, 25000 Besançon, France; 4 Université Marie et Louis Pasteur, Plateforme Exercice Performance Santé Innovation, 25000 Besançon, France; 5 Department of Movement and Sports Sciences, Ghent University, 9000 Ghent, Belgium; 6 Institute of Sport Sciences, University of Lausanne, 1015 Lausanne, Switzerland

## Abstract

Running biomechanics has long questioned whether a universally optimal running pattern exists. Current evidence indicates that no single technique is optimal for all individuals. Instead, runners self-optimize their movement according to anatomical structure, neuromuscular capacity, experience, and environmental constraints. This narrative review first examines the diversity of running patterns and the concept of self-organization, emphasizing that multiple biomechanical configurations can yield comparable running economy when aligned with individual characteristics. We then analyze how biomechanical models represent human locomotion. The spring–mass model explains key features of high-speed, elastic-driven running but becomes progressively limited as speed decreases. Actuator-based models incorporating active work and dissipation provide a more comprehensive account of endurance locomotion. From this modeling perspective, running mechanics are interpreted as a speed-dependent continuum from elastic-driven strategies at sprinting speeds to more muscle-driven strategies at slower speeds. Running speed is identified as the primary independent variable structuring these trends, while individual responses introduce variability around group-level norms. Translating this framework into practice requires individualized assessment rather than prescriptive technique modification. We present the PIMP method (Preferred running form assessment, Identification of inefficiencies in vertical load management, and Movement Plan individualization) as a structured approach to intervention. Central to this framework is the “Mind to Move” principle, in which outcome-focused imagery promotes biomechanical coherence between intrinsic capacities and external demands. The bouncy spring metaphor reinforces short elastic actions, whereas the rolling wheel metaphor supports prolonged, muscle-driven efforts. Performance optimization thus depends on context-sensitive, individualized strategies rather than replication of idealized forms.

**Supplementary Material:**

The online version contains supplementary material available at https://doi.org/10.1007/s40279-026-02449-w.

## Key Points

**Table:** 

Running technique is individualized, not universal. Each runner naturally develops a style that fits their body, strengths, and context. Embracing this individuality—rather than copying elite techniques—leads to better efficiency and fewer injuries
Running involves a continuum of movement strategies. An elastic-driven strategy dominates at sprinting speeds, using tendons for rebound, while a muscle-driven strategy becomes more important at endurance speeds, relying on muscular contraction. Training and technique should align with where the runner falls on this spectrum
Mind to move: achieving biomechanical coherence by aligning mental and physical execution. Lasting improvements come from internal intention and vivid imagery—like picturing yourself as a bouncing spring or a rolling wheel—which help the body adjust naturally and efficiently without overthinking mechanics

## Is There a Universally Optimal Running Pattern?

The notion that a single, universally optimal running pattern exists for all runners is unlikely due to the complexity of individual biomechanics. Rather than converging on a singular ideal, runners typically develop their own optimal styles based on their unique physical characteristics [1, 2]. Research shows that no one-size-fits-all running pattern exists, and personalized approaches are more effective.

Research supports the idea that runners self-optimize their biomechanics to achieve the most efficient running gait for their specific conditions [3, 4] and have a preferred/habitual movement path [5, 6]. For decades, studies have demonstrated that trained endurance runners often select stride lengths and frequencies that align closely with their optimal measures [7–13]. This phenomenon, known as self-optimization, suggests that runners subconsciously adjust their running pattern to minimize metabolic cost [4, 8, 14, 15]. More recent studies, such as those by Moore et al. [3], have identified U-shaped relationships between metabolic cost and variables like contact time and leg stiffness, reinforcing the idea that runners continuously refine their running pattern for efficiency.

Intrinsic factors such as anatomical structure, muscle strength, and flexibility significantly influence individual running styles. Recent research has focused on how these personal characteristics affect performance, running economy, and injury risk [16]. Machine learning and biomechanical analysis have revealed distinct functional groups among runners. For instance, Hoerzer et al. [17] identified eight functional groups based on biomechanical variables, while Phinyomark et al. [18] noted differences in knee angles that contributed to varied running styles. These findings highlight that variations in running patterns arise from differences in factors such as age, sex, and personal traits, reinforcing that there is no single optimal running pattern applicable to everyone.

Running patterns are inherently multifaceted and cannot be accurately described by a single variable. As early as 1985, Subotnick [19] emphasized that running patterns emerge from the interplay of several factors, including foot placement, arm swing, body angle, rear leg lift, and stride length. This view has been corroborated by subsequent research, which characterizes running as a global and dynamic system [20]. Importantly, these variables vary continuously rather than categorically, and span broad quantitative ranges across individuals and speeds, reflecting progressive modulation rather than discrete pattern shifts.

Researchers have nonetheless proposed descriptive categories to facilitate interpretation. For instance, “Groucho running” is defined by increased knee flexion and extended ground contact time [21], while “Pose running” is characterized by aligned acromion, greater trochanter, and lateral malleolus, along with brief ground contact time [22, 23]. “Chi running” features a midfoot or forefoot strike, short stride length, and a slight forward lean [24]. Similarly, “aerial runners” exhibit extended lower limbs during ground contact and a forefoot or midfoot strike pattern, whereas “terrestrial runners” display pronounced lower limb flexion during contact and a rearfoot strike pattern [20, 25]. However, these labels represent regions along a spectrum rather than mutually exclusive categories.

Recent methodological advances further support this continuum perspective. Machine-learning approaches have identified key gait features without imposing predefined classes, highlighting gradual inter-individual variation in coordination patterns [26]. In line with this view, van Oeveren et al. [2] proposed describing running patterns using two temporal variables: step frequency and duty factor. The duty factor reflects the proportion of ground contact time within the stride [27, 28]. Their framework organizes runners into descriptive groups (“stick”, “bounce”, “push”, “hop”, and “sit”) while explicitly acknowledging that these configurations lie along a continuous multidimensional space [2]. Notably, despite marked biomechanical diversity, different running patterns can yield comparable running economy when aligned with the individual’s physiological and morphological characteristics [4, 29–31].

These findings suggest that running efficiency does not stem from a single, universal gait pattern but rather can emerge from multiple strategies tailored to individual biomechanical characteristics. Rather than converging on a uniform technique, runners develop personalized gait solutions that reflect their unique biomechanical profiles. This diversity in economical patterns underscores the importance of individualized approaches to training and performance optimization, emphasizing that the most effective running form is one tailored to the individual’s needs.

## Can Running Patterns be Effectively Modeled?

Scientists have long relied on models to distill and represent the complexity of natural phenomena. In biomechanics, and particularly in running biomechanics, this approach is indispensable given the highly integrated and dynamic nature of human movement [32]. Running is not just a repetitive motion; it involves coordinated interactions among muscles, tendons, bones, and neural control systems, with forces generated, absorbed, and redistributed at every step. The challenge for researchers is to simplify this complexity into tractable frameworks without losing the essential mechanical principles that govern movement.

To address this challenge, biomechanical modeling deconstructs locomotion into fundamental components while preserving their functional relationships. Although the human body operates as an integrated system rather than as isolated parts, models allow specific mechanisms to be examined in isolation before being reinterpreted within the whole system. The objective is therefore not to replicate biological reality in full detail, but to capture the dominant dynamics that shape running behavior.

Within this context, the spring-mass model has emerged as a central framework in running biomechanics [33–41]. By representing the body as a point mass supported by a spring-like leg, it captures the essential mechanics of energy storage and return during stance. Despite its simplicity, this model has provided substantial insight into the mechanical organization of running patterns (see Electronic Supplementary Material for detailed formulation).

Building on this foundation, the spring-mass model has been widely used to interpret how runners interact with the ground. Nevertheless, its simplifying assumptions limit its ability to account for the full diversity of running behaviors observed in real-world conditions. In particular, certain movement characteristics deviate from the predictions of a purely elastic system [42–54], suggesting that additional mechanical processes are involved. To address these limitations, researchers have developed alternative modeling approaches incorporating active actuation and/or passive dissipation [55, 56]. These frameworks allow for a more comprehensive representation of running biomechanics, accounting for factors such as muscle-generated work and energy losses that are not captured by the traditional spring-mass model.

The simplest alternative is the actuator-only model, which represents the human body as a point-mass with legs modeled as massless, extendable actuators [55]. Unlike the spring-mass model, this framework does not rely on passive elastic energy storage. Instead, it focuses on minimizing muscle work through active control of leg length and orientation. This model is particularly useful for exploring how a runner can move efficiently from one place to another with the least amount of muscle exertion. By treating the legs as actuators that can be oriented, lengthened, and shortened, the actuator-only model provides a complementary perspective on gait selection, independent of elastic energy storage mechanisms like tendons. Notably, minimizing mechanical work alone was sufficient to reproduce both walking and running as optimal gaits, and at higher speeds, running emerged as a pseudo-elastic, bouncing-like movement even in the absence of passive elastic elements [55].

Building on these concepts, the actuator-spring-mass model represents another significant advancement [56]. This model extends the classic spring-mass framework by incorporating both active actuation and passive dissipation mechanisms. Although dissipation has been documented experimentally [57], it is often neglected in models of running energetics. The actuator in this model can perform both positive and negative work, compensating for energy losses occurring at foot–ground contact and due to tendon hysteresis. Notably, the model predicts that even when elastic energy return accounts for a substantial portion of the total positive work (~ 59%), active muscle work may still represent a large share of the metabolic cost (~ 68%), in agreement with experimental estimates [58]. In addition, the inclusion of an additional cost associated with rapid force development (force-rate) provides a plausible explanation for the smooth ground reaction force profiles observed in running, as well as for the presence of active negative muscle work [50, 59].

Compared with the purely conservative spring-mass model, the actuator–spring-mass model offers a more mechanistically faithful representation of running. It reproduces key empirical features, including braking–propulsion asymmetries, energy dissipation at touchdown, and speed-dependent increases in energetic cost. Its predictions across speeds and slopes are also consistent with known metabolic trends in humans [60, 61]. At the same time, the model remains a simplified representation, as it does not account for muscle–tendon moment arms, multi-joint dynamics, or detailed neuromuscular control. It should therefore be viewed as a foundational approximation rather than a comprehensive physiological model. Nonetheless, by explicitly integrating dissipation and force-rate penalties, it bridges a critical gap between theoretical biomechanics and experimentally measured energetics, providing a solid conceptual basis for translating modeling insights into applied contexts.

## Running Mechanics in Practice

The evolution of running biomechanics has been driven by both pioneering research and practical coaching innovations over the past decades. This section explores that trajectory—from the early 1950s investigations to contemporary insights into individualized running patterns.

### Vertical Rebound: Emergence of the Stretch–Shortening Cycle

In the early 1970s, seminal work by Thys and Margaria provided foundational insights into the mechanics of human movement [62]. Building on earlier theories by Hill [63, 64], who demonstrated that stretching a contracted muscle leads to the storage of elastic energy, Thys and Margaria investigated how this property influences movement efficiency. They examined a squatting exercise performed under two distinct conditions: (1) a rebound condition, in which leg extension immediately followed flexion, and (2) a no-rebound condition, in which a pause was introduced between phases. Their findings revealed that in the rebound condition, elastic energy stored during the eccentric phase contributed to greater movement velocity and mechanical efficiency in the subsequent concentric phase. Similarly, Asmussen and Bonde-Petersen [65] reported that rebound knee flexions had significantly higher efficiency (~ 40%) than non-rebound knee flexions (25%). These results highlighted the significance of a short coupling time—the duration between eccentric and concentric phases—for maximizing the contribution of stored elastic energy. If this transition is delayed, much of the stored energy dissipates as heat rather than contributing to mechanical work. This principle was considered especially relevant to activities such as jumping and running, where rapid, cyclical motions allow for optimal utilization of the stretch-shortening cycle.

Subsequent studies compared counter-movement jumps and squat jumps to further evaluate the functional implications of the stretch-shortening cycle. The counter-movement jumps, characterized by a preparatory downward movement, consistently produced higher jump heights and greater force output than the squat jumps, which begin from a static squat position. This performance advantage in the counter-movement jumps has been attributed to a more effective elastic energy return, as well as neural mechanisms such as potentiation. Thys et al. [66] demonstrated that in rhythmic jumping tasks involving small amplitude movements, up to 60% of the positive mechanical work was derived from stored elastic energy, underscoring the energetic efficiency of elastic recoil mechanisms. Building on this, Bosco et al. [67] quantified the biomechanical benefits of the counter-movement jumps over the squat jumps, reporting a 66% increase in average concentric force and an 81% increase in mechanical power. These enhancements were most pronounced when the coupling time was minimized, further reinforcing the importance of rapid transition between muscle stretching and shortening phases. Their findings emphasized that the stretch-shortening cycle effectiveness depends not only on the presence of elastic components, but also on variables such as pre-stretch velocity, muscle-tendon stiffness, and the timing of force development. Collectively, this body of research laid the groundwork for understandings of elastic energy utilization in ballistic and cyclic movements.

### Transfer to Sprinting: Embracing the Stretch-Shortening Cycle

The principles of the stretch-shortening cycle, initially observed in vertical tasks, quickly found relevance in horizontal locomotion, particularly in sprinting. Prior to the widespread adoption of the stretch-shortening cycle concept in training methodologies, sprinting technique was largely centered on a muscular “push-off” strategy, with athletes instructed to strike the ground with the heel and roll forward [68]. However, as the understanding of stretch-shortening cycle deepened, a paradigm shift occurred: the sprint stride was reconceptualized as a rebound, where rapid loading and unloading of the muscle-tendon units generated propulsion. This was most evident in sprinting events (100 m and 200 m), where elite athletes exhibit extremely short ground contact times (≤ 100 ms; Table 1) [69]. Such brief contact durations are biomechanically consistent with a forefoot strike pattern and result in very short coupling times, thereby optimizing elastic energy return. This biomechanical evolution coincided with innovations in track surfaces—most notably the introduction of tartan tracks, a viscoelastic material first used in the 1968 Mexico City Olympic Games [70]. These stiffer surfaces enhanced the efficiency of elastic rebound, reinforcing the shift from a push-driven to a spring-driven model of sprint performance.

**Table 1:** Performance data for men’s world record holders across running distances from 100 m to 160 km

Race	World record for men (year)	Time (hh:mn:ss.ms)	Speed (m/s) | (km/h)	Contact time (ms)
100 m	Usain Bolt (2009)	9.58	10.44|37.58	~ 90
200 m	Usain Bolt (2009)	19.19	10.42|37.52	~ 90
400 m	Wayde van Niekerk (2016)	43.03	9.30|33.47	~ 110
800 m	David Rudisha (2012)	1:40.91	7.93|28.54	~ 140
1000 m	Noah Ngeny (1999)	2:14.20	7.45|27.28	~ 150
1500 m	Hicham El Guerrouj (1998)	3:26.00	7.29|26.21	~ 160
3000 m	Jakob Ingebrigtsen (2024)	7:17.55	6.86|24.68	~ 160
5000 m	Joshua Cheptegei (2020)	12:35.36	6.63|23.83	~ 165
10,000 m	Joshua Cheptegei (2020)	26:11.00	6.36|22.91	~ 170
Half marathon (21.1 km)	Jacob Kiplimo (2025)	56:42.00	6.20|22.33	~ 170
Marathon (42.2 km)	Kelvin Kiptum (2023)	2:00:35.00	5.83|21.00	~ 180
50 km	Clayton Jordan Albertson (2022)	2:38:43.00	5.25|18.90	~ 185
100 km	Aleksandr Sorokin (2023)	6:05:35.00	4.56|16.41	~ 190
160 km (100 miles)	Aleksandr Sorokin (2022)	10:51:39.00	4.10|14.76	~ 210

The table includes official record times, average running speeds, and ground contact times. These values illustrate the variation in performance and mechanical demands across sprinting, middle-distance, and ultra-endurance events. Record times were obtained from https://en.wikipedia.org/wiki/List_of_world_records_in_athletics and https://en.wikipedia.org/wiki/Ultramarathon for the 100-mile distance. Websites were visited on 2 April 2025. Given the established correlation between contact time and running speed [84], ground contact times (force plate measurements) were obtained from Nummela et al. [85] at speeds corresponding to each performance level

This transformation in sprint technique was accompanied by refined biomechanical strategies that emphasized elastic energy utilization, impulse management, and minimization of braking forces. Efficient sprinting requires not only forefoot contact but also an active backward pull of the foot at touchdown, often referred to as the “griffe” or “clawing” effect, which aligns horizontal foot velocity with the body’s forward speed to minimize deceleration [71, 72]. The stretch-shortening cycle allows muscles to absorb force during the eccentric phase and release it explosively in the concentric phase, a mechanism that plyometric training further enhances [69, 73]. Recent studies have validated these early statements, showing that as steady-state running speed advances toward maximum sprinting, the human ankle plantar-flexors increasingly prioritize the storage and recovery of tendon elastic strain energy over muscle fiber work, confirming the central role of passive elastic structures in sprint propulsion [74]. The importance of core and pelvic stability has also been recognized: a well-positioned pelvis improves force transmission and reduces energy leakage from lateral or rotational movements [75]. At maximal velocity, the focus shifts from acceleration to maintaining speed, which is achieved by reducing ground-contact time, aligning foot placement under the center of mass, and optimizing stride variables. The arms and upper body, although often overlooked, contribute rhythm and counterbalance, aiding in efficient force transfer and coordination. Together, these insights mark a departure from simplistic force application strategies toward a more integrated, elastic, and efficient model of sprint performance—where the rebound is not only a biomechanical advantage but a central tenet of sprinting.

### Emergence of a Unique Model: The Spring-Mass Model

The biomechanical insights gained from previously mentioned studies on jumping and sprinting—particularly the role of the stretch-shortening cycle and the optimization of ground contact mechanics-paved the way for the conceptualization of a unifying model in human locomotion: the spring-mass model [33–41]. As introduced earlier, this framework describes locomotion as the interaction between a point mass and a spring-like leg, in which leg stiffness and contact time regulate the bouncing trajectory of the center of mass. By emphasizing elastic energy storage and return, this model provided a coherent mechanical explanation for efficient high-speed running.

**Fig. 1:**
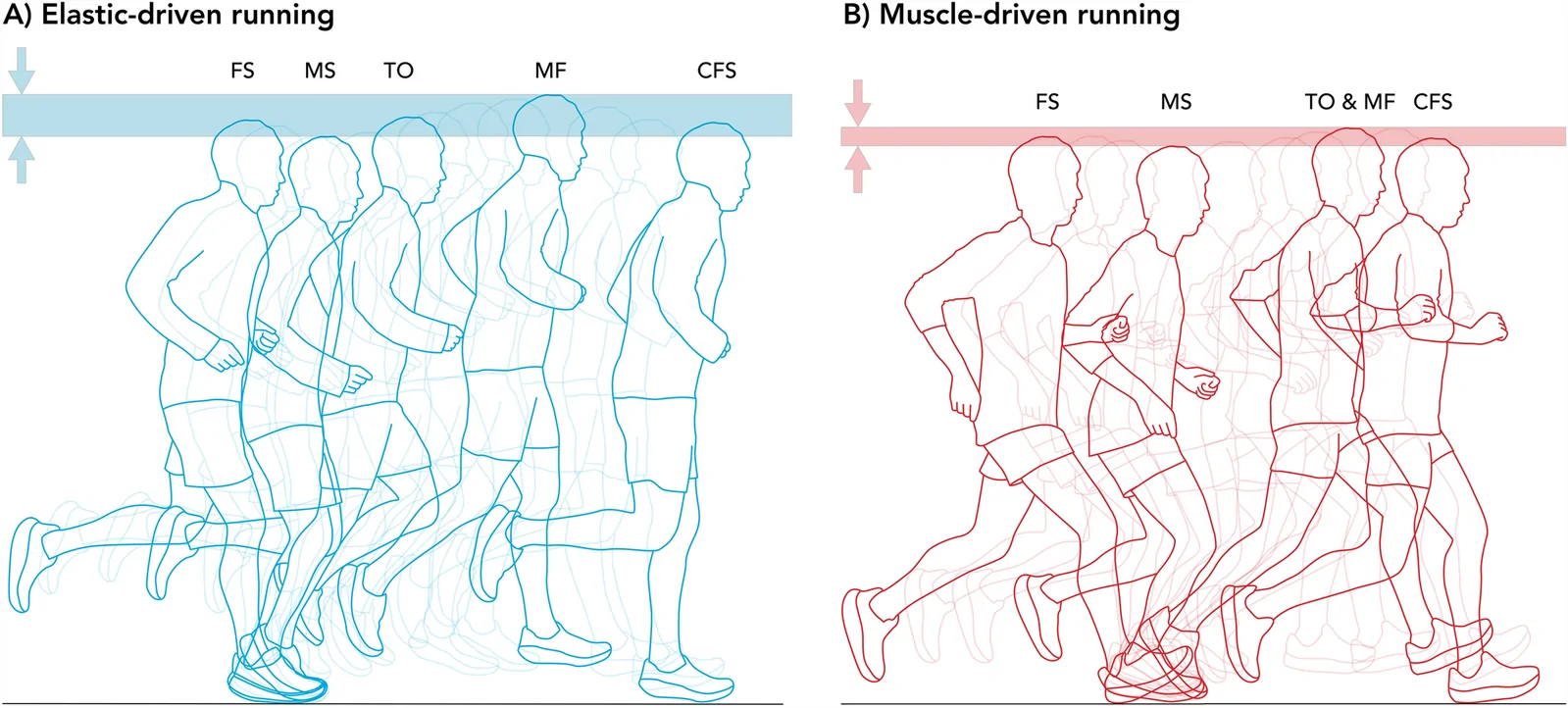
Representative kinematic traces of the two opposed running modes and their relative step‐time scaling. **A** (blue): Elastic‐driven running, featuring short contact time and long flight time, large vertical oscillation, symmetric ground contact phase, elevated leg stiffness, and predominant tendon recoil. **B** (red): Muscle‐driven running, characterized by long ground contact duration and short flight time, small vertical oscillation, asymmetric ground contact phase, large joint excursion, and more active muscle work. Bold outlines denote key running events: foot-strike (FS), mid-stance (FS), toe-off (TO), mid-flight (MF), and contralateral foot-strike (CFS). Shaded outlines show successive body positions in between the key events. The horizontal bars at the top of the figures illustrate the vertical oscillation

From the early 2000s onward, models inspired by spring-mass mechanics began to be applied to distance running, offering insights into energy-conservation strategies among endurance runners across all performance levels. Over time, the spring-mass model transcended the realm of research to shape running technique and coaching philosophies. Notably, adopting a forefoot or midfoot strike pattern contributes to achieving a more linear stiffness curve [76] and enhances energy storage in the plantar flexor tendons [77], albeit with an associated increase in ankle power requirements [78]. These biomechanical insights have significantly catalyzed the emergence of technique-driven approaches such as the Pose Method [22] and Chi Running [24]. These methodologies advocate for a light and rapid footfall, and an emphasis on falling forward into each stride, allowing gravity and elasticity to replace muscular effort as the primary sources of propulsion. These running techniques exemplify the degree to which the spring-mass model permeated both elite coaching in sprint practice and recreational training in endurance practice. Representative kinematic traces of such elastic-driven running style are depicted in Fig. 1A.

Hence, the spring-mass model became the dominant biomechanical framework for understanding running, promoting a conceptual shift toward viewing the runner not as a pusher of mass, but as an oscillating system tuned to recycle energy with each stride. Although vertical oscillation implies a certain energy cost—since the body must work against gravity—it is precisely this movement that enables the storage of elastic energy during ground contact, allowing runners to regain that energy “for free” in the push-off phase.

### Beyond the Spring-Mass Paradigm: Biomechanical Adaptations in Endurance Running

While the spring-mass model has long served as a robust framework for understanding the biomechanics of fast track and field disciplines—particularly sprinting and middle-distance events, where rapid force application and efficient elastic energy return are essential—its relevance diminishes as running speed decreases. In theory, the model is sufficiently flexible to describe low-speed locomotion characterized by a prolonged ground contact time [33]. However, empirical evidence shows that, as speed declines, whether due to longer distances or lower athletic levels, runners adopt a set of biomechanical strategies that increasingly diverge from the model’s foundational assumptions.

One notable shift is in foot-strike patterns. Whereas sprinters predominantly utilize a forefoot strike to capitalize on the stretch-shortening cycle [79, 80], endurance runners often adopt a rearfoot strike. This transition is not merely anecdotal; more than 85% of recreational runners exhibit a rearfoot strike in distance races above 10 km (Table 2; see Bovalino and Kingsley [81] for a complete review). This transition also applies to elite runners; 75% were rearfoot strikers at the 2004 Sapporo International Half Marathon in Japan [82], and more than 54% of marathoners adopted a rearfoot strike pattern at the 2017 IAAF World Championships [83]. This widespread transition toward rearfoot striking alters the mechanical behavior of the lower limb, resulting in a stiffness curve that deviates from the linear profile predicted by the spring-mass model.

**Table 2:** Estimated performance and biomechanical characteristics of male recreational runners across distances ranging from 100 m to 160 km

Race	Time (hh:mn:ss)	Speed (m/s)|(km/h)	Contact time (ms)	Rearfoot strikers (%)
100 m	20	5.00|18.0	~ 190	~ 0
200 m	40	5.00|18.0	~ 190	~ 0
400 m	1:32	4.35|15.7	~ 210	~ 0
800 m	3:33	3.76|13.5	~ 230	–
1500 m	7:13	3.46|12.5	~ 250	–
3000 m	15:13	3.29|11.8	~ 250	–
5000 m	31:28	2.65|9.6	~ 295	–
10,000 m	57:15	2.91|10.5	~ 275	~ 96
Half marathon (21.1 km)	1:59:48	2.94|10.6	~ 275	~ 89—95
Marathon (42.2 km)	4:14:29	2.76|10.0	~ 285	~ 89—94
50 km	5:00:32	2.77|10.0	~ 285	~ 85
100 km	12:07:48	2.29|8.2	~ 320	–
160 km (100 miles)	22:35:46	1.98|7.1	~ 330	~ 89

The table includes average completion times and speeds, ground contact times, and prevalence of rearfoot strikers. These values illustrate the variation in performance and mechanical demands across sprinting, middle-distance, and ultra-endurance events. 100-m to 3-km average times were obtained using a 40-year-old man and a 50% grade using the reverse age grading calculator available here: https://runbundle.com/tools/reverse-age-grading-calculator, and similarly for 50- to 160—km average times using the calculator: http://www.howardgrubb.co.uk/athletics/mldrroad25.html. 5 km to marathon average times were given as the 50th percentile as reported in https://runrepeat.com/how-do-you-masure-up-the-runners-percentile-calculator. Websites were visited on 15 April 2025. Given the established correlation between contact time and running speed [84], ground contact times (force plate measurements) were obtained from Nummela et al. [85] and Malisoux et al. [86] at speeds corresponding to each performance level. Sprinters were considered as forefoot strikers [79, 80]. To the best of the authors’ knowledge, no study has reported the foot-strike pattern during track and field events. Prevalences of rearfoot strikers were obtained from Murray et al. [132] for 10 km, from Larson et al. [133] and Latorre-Román et al. [134] for half marathon, from Larson et al. [133] and Kasmer et al. [135] for marathon, from Kasmer et al. [136] for 50 km, and from Kasmer et al. [137] for 160 km events. For a complete review of foot-strike patterns during overground distance running, see Bovalino and Kingsley [81]

Additionally, contact times lengthen in endurance running for both elite and recreational runners (Tables 1, 2), contrasting with the brief, forceful contacts observed in sprinting [84–87]. This prolongation of ground contact time inevitably increases the coupling time, thereby reducing the efficiency of elastic energy return and shifting the burden toward active muscle work. As coupling time increases, the ability to exploit the stretch-shortening cycle diminishes, further distancing the mechanics of long-distance running from those observed in fast, spring-like locomotion.

Moreover, the temporal and spatial symmetry between braking and propulsive phases of ground contact time—a hallmark of the spring-mass model—becomes less pronounced with decreasing speed [42]. This evolving asymmetry reflects a broader shift in the mechanical behavior of the muscle-tendon unit across different running velocities, pointing to a speed-dependent interplay between muscle and tendon contributions to mechanical work [42]. At lower speeds, active muscle fibers undergo more significant length changes, indicating a greater reliance on muscular effort for both shock absorption and propulsion. However, as running speed increases, muscle activation intensifies and fibers increasingly resist elongation. Consequently, a larger proportion of the stretch imposed on the muscle-tendon unit is absorbed by the tendinous structures. As suggested by Cavagna [42], around speeds exceeding ~ 14 km/h, muscle fibers approach an almost isometric state, allowing tendons to undergo most of the length change during the stretch-recoil cycle. In such cases, the muscle–tendon unit behaves more like a passive spring, closely aligning with the assumptions of the spring-mass model. Despite this spring-like behavior, some active work from the contractile elements remains necessary to compensate for energy losses due to tendon hysteresis—a phenomenon that, while present, contributes minimally to overall mechanical inefficiency [88–90].

Recent in vivo investigations combining ultrasound imaging and musculoskeletal modeling provide more direct insight into this speed-dependent modulation of muscle–tendon unit behavior. Across increasing running speeds, the ankle plantar flexors exhibit a progressive shift toward reduced fascicle shortening velocity and greater reliance on elastic tissue deformation. Both Lai et al. [91] and Monte et al. [92] demonstrated that, at faster speeds, muscle fascicles operate closer to quasi-isometric conditions while the series elastic elements undergo larger and more rapid stretch-recoil cycles. In these conditions, a greater proportion of positive mechanical work is attributable to elastic strain energy, reinforcing the spring-like behavior predicted by the spring–mass model. Conversely, at slower speeds, fascicles undergo larger and more sustained length changes during stance, consistent with increased active contractile work and diminished elastic dominance.

Importantly, alterations in foot-strike pattern further illustrate that internal muscle-tendon unit behavior cannot be inferred solely from external kinematics. Swinnen et al. [93] showed that rearfoot and forefoot or midfoot strikers differ in fascicle-shortening velocity and muscle-force production during early stance, yet these differences were not accompanied by systematic changes in total series elastic element stretch. Moreover, simulated triceps surae metabolic energy expenditure did not differ substantially between strike patterns [94]. Together, these findings indicate a progressive reweighting of mechanical contributions within the muscle–tendon unit with decreasing speed, with elastic recoil remaining present but active muscular control becoming increasingly dominant.

### From Springs to Actuators: The Muscle-Driven Strategy

As running velocity decreases, the mechanical characteristics of human gait undergo a progressive transformation. The hallmark features of high-speed running—such as a distinct and extended aerial phase, brief ground contact time, and spring-like behavior of the lower limbs—gradually diminish. In their place emerges a running pattern dominated by active muscular control (muscle-driven) and characterized by a distinctly different set of biomechanical signatures. This form of locomotion, often referred to as grounded running [95], marks a clear departure from the spring-mass paradigm that aptly describes faster gait dynamics. Figure 1B depicts representative kinematic traces of the muscle-driven running style often employed in long-distance running.

At the core of endurance running lies an extended ground contact phase, reflected in a higher duty factor. In some cases, this shift even leads to the complete disappearance of a flight phase [96]. For example, observational studies indicate that 16% of participants in a large-scale open marathon [97] and 33% of runners in a 5-km recreational event [98] exhibited a grounded running pattern without any aerial phase, at speeds ranging from 5.5 to 10.4 km/h. This adaptation reflects a strategy that emphasizes continuous support over ballistic exchange, replacing the rebound mechanics of aerial running with a more sustained interaction with the ground.

The transition to muscle-driven running demands a more compliant limb behavior [95], where compliance is defined as the inverse of leg stiffness. Grounded running is characterized by reduced effective leg stiffness, lower vertical oscillation of the center of mass, and enhanced impact attenuation [95]. The trajectory of the center of mass no longer follows a pronounced vertical arc but instead progresses forward with a more restrained vertical excursion [95]. During stance, the limb exhibits a more sustained force-time profile with prolonged ground contact, allowing controlled compression and progressive extension as the body moves forward. Importantly, this reduction in vertical stiffness does not necessarily require greater knee flexion [96], but reflects modified loading dynamics and redistribution of mechanical work throughout stance. Rather than relying predominantly on rapid elastic recoil, propulsion emerges from a greater relative contribution of active muscle work coordinated across joints. The running gait becomes less about vertical rebound and more about forward progression.

Styles such as terrestrial [20] and grounded [96, 99] running all share key characteristics: reduced vertical displacement, prolonged ground contact, and a visible lowering of the runner’s posture—often giving the impression of “sitting into the stride”. Similarly, the concept of high duty factor running [100, 101] quantitatively captures these traits, identifying runners who spend a greater portion of each stride in contact with the ground and tend to favor a rearfoot strike pattern with high limb compliance. The aggregate visual signature of this running style includes a flatter torso trajectory, visibly deeper leg angles during mid-stance, and smoother, rolling transitions from step to step. It is a controlled, deliberate gait that privileges contact time and postural management over spring-like rebound. The entire body appears to remain more grounded, anchored to the surface, rather than springing away from it.

This shift in mechanics also carries broader implications. Grounded running is associated with reduced impact loading, lower peak ground reaction forces, and diminished stresses on muscle–tendon units [95, 96, 99]. These characteristics make it particularly relevant for certain populations, including older adults and individuals with higher body mass index—groups who may benefit from a running style that mitigates mechanical strain. The muscle-driven strategy of endurance running thus not only exemplifies a biomechanical alternative to traditional aerial gait, but also represents a strategic adaptation with potential relevance across diverse physiological profiles.

### The Transition Zone: Coexistence of Elastic- and Muscle-Driven Strategies

At the extremes of locomotor demand—either elastic-driven sprinting or muscle-driven endurance running—only those that are biomechanically adapted survive. This is exemplified by the absence of heel striking among elite 100-m sprinters and its prevalence in over 90% of runners competing at the 100-mile distance (Table 2). Between these poles exists a transitional biomechanical zone, typically occurring around 12–16 km/h, where neither the elastic-driven nor the muscle-driven paradigm is fully dominant. Within this intermediate range, runners exhibit a hybrid locomotor strategy, wherein both tendinous elasticity and active muscular force production contribute substantially to propulsion. The precise inflection point of this transition is subject to individual variability, shaped by factors such as anatomical structure, musculoskeletal morphology, neuromuscular coordination, and training history.

Taken together, these observations support the notion of a continuum between two distinct mechanical paradigms: an elastic-driven strategy predominating at sprinting running speeds, and a muscle-driven strategy becoming more relevant at endurance speeds. When speed exceeds ~ 14 km/h, the muscle-tendon unit operates increasingly like a passive spring, with minimal muscle fiber length change compared to tendon elongation and efficient elastic recoil, as posited by the spring-mass framework [42]. In contrast, slower speeds are marked by longer ground-contact times, greater braking-propulsion asymmetry, and substantial muscle fiber involvement, reflecting a shift toward active, contractile work [42]. Around this transitional threshold, both strategies may coexist, with tendons and muscles sharing the mechanical load in a more integrated manner.

Importantly, running speed should be considered the primary independent variable structuring these mechanical trends. At the group level, increases in speed are consistently associated with shorter contact times, lower duty factors, greater peak ground reaction forces, higher vertical stiffness, etc. [86]. However, individual adaptations to speed are not uniform. Detailed analyses of foot-strike angle across running speeds show that, although group means suggest a gradual shift away from rearfoot striking, some runners maintain a stable strike pattern across speeds, whereas others progressively modify their foot–ground interaction as velocity increases [102]. Moreover, the relative positioning of individuals within the distribution of spatiotemporal variables is only partially preserved across speeds [103]. For instance, while certain runners consistently exhibit comparatively high or low duty factors across a broad velocity range, others markedly change their relative ranking as speed increases. Consistency therefore decreases as speed differences widen. This indicates that speed defines the normative direction of biomechanical change at the population level, yet the magnitude and structure of adaptation depend on each runner’s initial coordination pattern. Accordingly, biomechanical variables (e.g., foot-strike pattern, duty factor, etc.) should not be interpreted as independent drivers of muscle–tendon behavior without first accounting for the primary effect of speed and its interaction with individual baseline mechanics. This also implies that externally visible technique features cannot be universally prescribed without considering how each runner responds to speed-dependent mechanical demands.

Rather than conforming to a universal technique, runners tend to self-optimize their gait based on energetic efficiency and fatigue resistance—especially within this transitional speed zone [3, 4]. Current evidence provides limited support for interventions aimed at altering foot-strike patterns [104]. Although forefoot striking has gained popularity in specific contexts, no single technique has been conclusively demonstrated to confer universal performance or injury-prevention benefits [4, 104]. Claims that an efficient stride is characterized by pronounced vertical oscillation and forefoot landing are not substantiated by empirical evidence [105]. Instead, substantial inter-individual variability emerges [2, 20, 101]. Some runners adopt an elastic strategy—characterized by a springy gait and forefoot strike—while others employ a more muscular strategy, marked by flatter trajectories and rearfoot contact. Yet, despite these kinematic differences, the associated metabolic cost remains remarkably similar when performance level is controlled. Multiple studies have shown that running economy does not significantly differ across foot-strike patterns or more global running gaits [4, 29–31, 100, 106], supporting the view that running technique should be individualized rather than universally prescribed.

This underscores a critical insight: running efficiency is not dictated by the external form of movement, but by how well a given pattern integrates with an individual’s physiological and mechanical characteristics. Research consistently shows that runners intuitively adopt strategies that minimize oxygen consumption and perceived effort—patterns refined over years of neuromotor adaptation and exposure to task-specific demands [81, 105]. While some argue—based on findings by Burns et al. [107]—that elite runners more closely align with spring-mass mechanics, demonstrating greater symmetry in ground reaction forces and consistent elastic behavior even at submaximal speeds, such interpretations must be made cautiously. In that study, elite athletes were ~ 20 kg lighter than recreational counterparts, highlighting a potential selection bias and reinforcing the influence of morphology/anatomy in promoting spring-like mechanics.

Recognizing and respecting this diversity is essential in applied practice. Rather than imposing a standardized “optimal” running gait, coaches and clinicians should prioritize individual assessments and encourage adaptations that align with each runner’s unique biomechanical and physiological profile—within the boundaries of known efficient running models (elastic-driven or muscle-driven strategies). This approach does not advocate for unrestricted self-expression but rather encourages evidence-based personalization, ensuring that individual variability is supported by sound mechanical principles. Such a framework fosters not only performance optimization but also long-term resilience and injury prevention.

Still, efficient running is not merely the result of external form; it emerges from the subtle, individualized adaptations shaped over time through practice, perception, and self-organization. To truly understand and enhance performance, we must go beyond the mechanics alone and consider how runners coordinate their bodies in dynamic environments—guided not only by physics, but by perception, intention, and experiential learning.

## Applying Biomechanical Coherence and the Mind to Move Framework

### Self-Optimized Running Patterns: The Body Finds Its Own Way

Humans are inherently self-optimizing organisms. While emulating the biomechanics of elite runners might appear logical, such imitation often conflates cause and effect—copying performance outcomes without understanding the underlying adaptations. Rather than prescribing an idealized elastic-driven running style based on the spring-mass model, a more effective strategy embraces what is termed the PIMP approach (Preferred running form assessment, Identification of inefficiency in vertical load management, and Movement Plan individualization), which integrates internal, individualized variables and respects the runner’s spontaneous running pattern [108]. This paradigm supports the view that self-optimization, not mimicry, is the foundation of biomechanical efficiency. As runners accelerate, their gait may naturally evolve toward spring-mass-like behavior; however, this shift is best understood as an emergent adaptation to both the runner’s intrinsic biomechanical dispositions and the external demands imposed by running speed—not as the outcome of an imposed or consciously rehearsed technique.

Identifying the most effective running pattern is a personalized process, shaped by biomechanical profile, individual goals, and environmental context. There is no single “correct” way to run. Instead, performance and injury prevention are best supported by understanding and refining the runner’s current strategy. Whether aiming to run faster or farther, meaningful technical changes stem from biomechanically informed, goal-specific interventions.

A practical tool for guiding this personalization is by evaluating and placing the running pattern along a terrestrial-aerial continuum [20]. This is operationalized through the PIMP framework [108], a three-step process:

1. P—profile the runner’s spontaneous running pattern (preferred running form assessment)
2. I—identify inefficiencies in vertical load management
3. MP—map a personalized movement plan (movement plan individualization),

*Step 1: Profiling the Spontaneous Running Pattern* The process begins with assessing the runner’s habitual gai—often referred to as the spontaneous pattern—which reflects innate motor preferences shaped by musculoskeletal structure, muscle strength, neuromotor history, and prior training. Gait analysis (e.g., video assessment or professional consultation) helps characterize this baseline using key indicators such as foot-strike pattern (rearfoot, midfoot, forefoot), stride length, cadence, and postural tendencies (flexion vs. extension dominance). This observational step establishes the runner’s position on the terrestrial–aerial continuum, providing essential context for interpreting mechanics. Quantitative positioning along this continuum has been operationalized elsewhere using the subjective Volodalen score [20, 25], which shows good intra- and inter-rater reliability [109]. As an objective alternative, duty factor can also be used to quantify the same aerial–terrestrial continuum and provides comparable subgrouping of spontaneous running forms [110]. This profiling also provides an initial expectation regarding how vertical load is likely managed during stance (i.e., toward a more compliant or more stiff strategy), which is examined explicitly in Step 2.

*Step 2: Identifying Inefficiencies in Vertical Load Management* In efficient, spontaneous running, observers often resort to coaching language—describing visible relaxation, fluid coordination, and natural rhythm. In contrast, inefficiency tends to manifest as tension, imbalance, excessive vertical oscillations, or uncontrolled movement in the frontal plane [111]. These observable qualities often reflect a deeper biomechanical reality: efficient running requires the body to manage vertical load effectively during stance, i.e., to absorb, transmit, and redirect vertical ground reaction forces through time and across joints. Within the PIMP framework, vertical load management is therefore defined as the runner’s ability to maintain a functional compromise between compliance (controlled joint deformation and force attenuation) and stiffness (resistance to deformation and effective force transmission).

Deviations from this functional compromise are categorized as either “too soft” (excessive compliance) or “too hard” (excessive stiffness). Systematic analyses [112, 113] indicate that many kinematic, kinetic, and spatiotemporal injury-related factors can be interpreted within this framework, such that most commonly reported risk markers reflect an overly compliant or overly stiff running pattern (see Table 1 in Gindre et al. [108]). For example, an increased vertical instantaneous loading rate is typically associated with a too-hard profile, whereas excessive peak hip adduction, often observed as contralateral pelvic drop, is more consistent with a too-soft pattern. Although these variables are most precisely quantified in laboratory settings using force platforms and motion capture systems, the underlying load-management tendencies they represent can often be inferred in applied contexts. Structured visual observation and, when available, lower-cost technologies such as wearable sensors or video-based analyses allow practitioners to identify and characterize inefficiencies in the vertical load management. Importantly, both too-soft and too-hard profiles may increase tissue-specific stress through different mechanical pathways, which helps explain why apparently opposing patterns may contribute to the same injury type [114], reinforcing the need for individualized interpretation rather than categorical generalizations.

To illustrate this point, two runners may present with similar symptoms and clinical findings consistent with plantar fasciopathy, yet display different inefficiencies in vertical load management. In one case, the injury occurs by excessive pronation and a more compliant stance behavior (too soft). In the other case, the injury is caused by an increased impact intensity (too hard) [114]. Within the PIMP framework, these cases would not lead to the same intervention direction, as the movement plan is individualized according to the identified load-management bias, thereby guiding Step 3.

*Step 3: Mapping a Personalized Movement Plan* The final stage involves designing a targeted conditioning program tailored to the runner’s profile. Extension-based exercises (e.g., plyometrics) enhance stiffness, reduce ground contact time, and promote vertical lift and forward propulsion of the center of mass, fostering alignment of the body (shoulder–hip–knee–ankle). Flexion-based exercises (e.g., squats) prolong ground contact, encourage joint mobility, and shift the center of mass backward and downward, emphasizing controlled compliance. The runner’s natural pattern—whether more aerial (stiff and vertical) or terrestrial (compliant and horizontal)—guides this selection. Runners with a too-hard profile benefit from flexion-oriented exercises to absorb impact forces more effectively, while those who are too soft require extension-dominant training to reinforce propulsion and structural alignment [108]. Importantly, these orientations—toward flexion or extension—should not be interpreted as rigid prescriptions but as directional tendencies, adjusted progressively and by degrees. The goal is not to impose dramatic biomechanical shifts but to guide the athlete’s system toward greater efficiency by building on their existing pattern. This aligns with the principles of differential learning [115], which emphasize variability around the individual’s natural coordination solution. By exploring movement variability within a defined direction of change, runners can adapt more organically, reduce inefficiencies, and foster robust, self-regulated performance adaptations.

Together, these three steps provide a structured framework for reinforcing an optimal running pattern by integrating clinical experience, biomechanical data, and strength conditioning principles to develop personalized exercise plans. By combining running style assessment, evaluation of vertical load management, and individualized movement planning, the PIMP framework offers a structured way to align mechanical strategy with the runner’s current capacities and goals.

Importantly, this individualized perspective is consistent with epidemiological and biomechanical evidence linking biomechanical variables to running-related injury risk [112, 116, 117]. Systematic reviews have reported associations between running-related injuries and spatiotemporal, kinematic, and kinetic variables, including loading rate, joint kinematics, contact time, and leg stiffness [112, 116]. For example, Malisoux et al. [117] identified duty factor as a context-dependent risk factor in recreational runners, showing that a lower duty factor was associated with a higher injury risk, particularly when combined with softer footwear. Within the PIMP framework, such findings support the interpretation of duty factor and related variables not as isolated predictors, but as indicators of how mechanical load is distributed over time and managed relative to individual tissue capacity and running style. Ensuring long-term performance and injury resilience thus requires maintaining coherence between internal mechanical capacities and the external demands imposed by the running environment.

### Biomechanical Coherence: Aligning Internal Capacities with External Demands

#### Selecting External Conditions that Match the Running Pattern

One primary strategy for achieving biomechanical coherence is to select external conditions that align with a runner’s natural coordination profile. Instead of modifying the body to meet external demands, this approach focuses on optimizing performance by choosing environments that suit the runner’s spontaneous movement strategy [2, 104]. For instance, assuming equivalent energetic capacities between elastic- and muscle-driven runners, the former, characterized by spring-like, aerial mechanics, generally perform best in shorter events on firm and reactive surfaces such as track or asphalt, typically using stiff-soled shoes. Their training often includes extension-based exercises to promote vertical propulsion, reduce contact time, and enhance body alignment. In contrast, muscle-driven runners, who display more compliant, terrestrial mechanics, are typically better suited to longer distances, uneven or softer terrain like trails or sand, and flexible footwear. Their training benefits from flexion-based exercises that increase joint range of motion, lengthen ground contact time, and support muscular propulsion. Although this rationale is biomechanically coherent and widely supported in practice, empirical evidence confirming its specific effectiveness remains limited and warrants further investigation.

By tailoring race formats, surfaces, footwear, and exercise selection to the runner’s intrinsic motor pattern, this strategy enables the body to operate within its preferred biomechanical constraints, enhancing efficiency and reducing the risk of injury [6]. A comparable concept was previously proposed in relation to the natural running patterns observed in young soccer players [118]. In addition, previous research has shown that the biomechanical features of aerial and terrestrial running styles are linked to subjective feelings of pleasure and displeasure. These affective responses vary with external factors such as running speed. Runners tend to experience greater pleasure under conditions that enhance their biomechanical efficiency—for instance, individuals with shorter ground-contact times and longer flight times report more positive feelings when running at higher speeds. Since locomotor performance involves trade-offs between an individual’s biomechanical traits and environmental constraints, these affective patterns reflect an intrinsic alignment between movement efficiency and perceived comfort [119].

#### Modifying Internal Mechanics to Meet External Demands

Alternatively, a runner may choose to engage in activities or pursue goals that do not naturally align with their default pattern—for example, a muscle-driven runner targeting high-speed track performance or an elastic-driven runner preparing for an ultra-trail race. In such cases, biomechanical coherence remains attainable because the human body possesses a remarkable capacity for adaptation [120]. However, aligning movement patterns with the demands of the chosen environment requires the progressive and structured modification of intrinsic factors such as strength, coordination, and tissue tolerance.

This adaptation process often includes targeted technical drills, strength training, or physiological changes such as body composition adjustments. For example, a runner with limited rebound might start by gradually accelerating their foot roll and developing forefoot loading capacity, progressively moving toward a spring-mass-like gait. Similarly, a heavier runner aiming to improve aerial mechanics might work on vertical force production and reduce body mass to facilitate elastic behavior. Evidence from Burns et al. [107] indicates that elite athletes, who were ~ 20 kg lighter than their recreational peers, demonstrated greater elastic behavior, highlighting how morphology can favor spring-like mechanics.

Crucially, these changes should be introduced gradually and should be grounded in a clear understanding of the runner’s baseline coordination profile. Attempting to shift too far from one’s innate coordination strategy—too quickly or too drastically—can lead to inefficient movement patterns and increased injury risk. Sustainable adaptation begins with a clear understanding of one’s baseline and progresses through manageable, targeted interventions that expand biomechanical capacity while respecting individual limits [120].

Recent epidemiological findings reinforce the importance of this progressive approach. Sudden increases in mechanical load have been shown to induce an immediate elevation in running-related injury risk [121], indicating that injury risk is not solely governed by absolute load magnitude, but also by the rate at which external demands exceed the runner’s current adaptive capacity. Abrupt changes in training volume, intensity, terrain, footwear, or technique may transiently disrupt the runner’s established coordination solution, forcing rapid reorganization of internal mechanics under constrained conditions. In this light, adaptation can be understood as the system’s capacity to reorganize efficiently when external demands shift—a process that depends not only on tissue tolerance and strength, but also on how movement is internally represented and regulated.

#### Mind to Move: Coherence Between Self and Goal

Ultimately, performance optimization is not purely biomechanical; it is also cognitive and perceptual. True coherence emerges when internal capacities—who I am, how I move, and how I feel—align with the external task—what I want to achieve, where and how fast I must do it. Neuroscientific work by Berthoz shows that movement planning begins with an internal simulation of the intended action, after which the body “fills in” the mechanics to match that goal [122]. We refer to this alignment of mental intention and mechanical execution as the “mind to move” principle: change must start from the inside, with thought and sensation guiding the body’s adjustments.

**Fig. 2:**
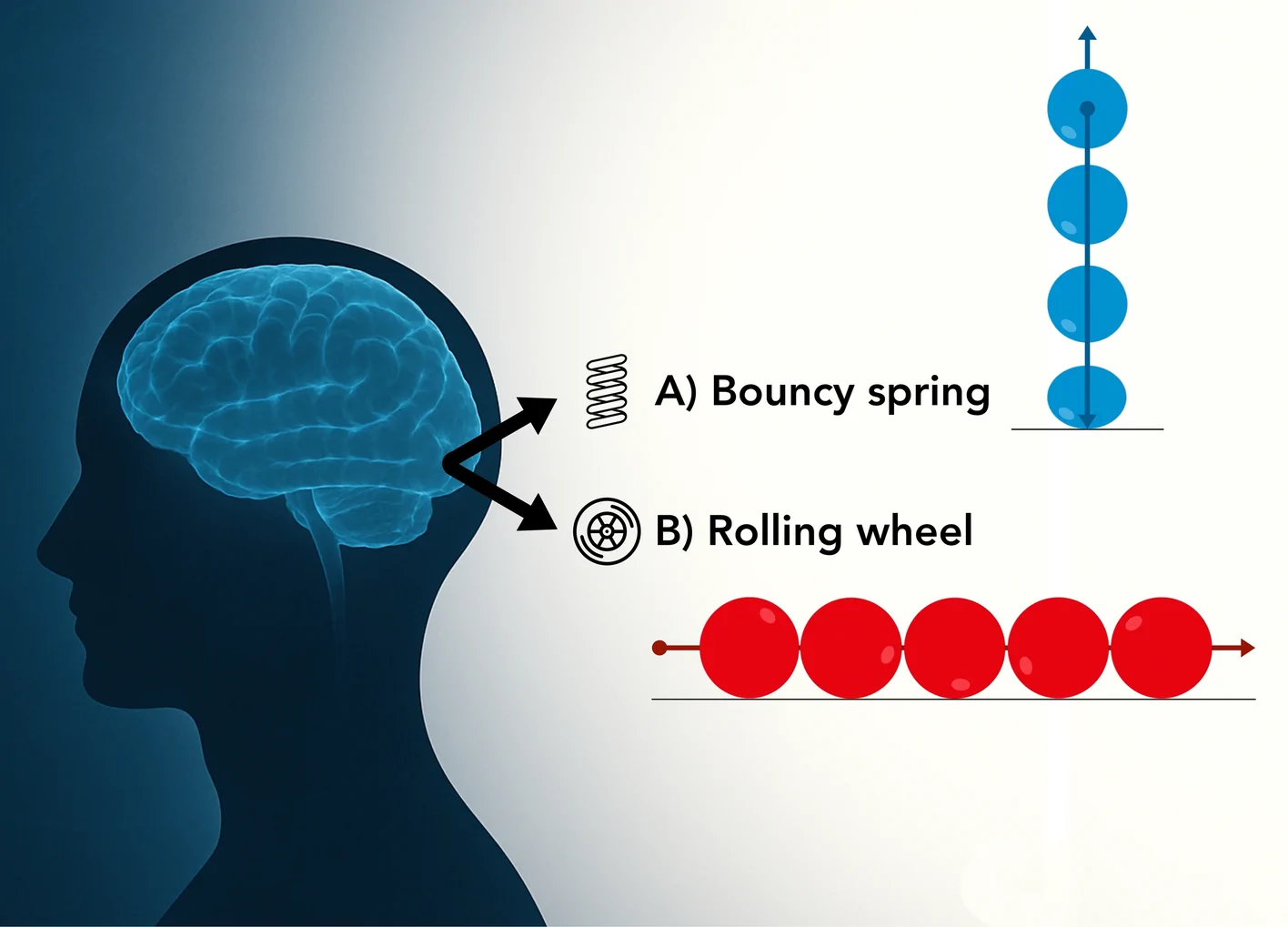
Schematic depiction of two intuitive externally oriented mental imagery, (1) the “bouncy spring” for short elastic actions, and (2) the “rolling wheel” for long enduring efforts, used in motor‐learning interventions. Athletes cued with the spring image instinctively increase leg stiffness, shorten ground‐contact time, and adopt an upright, dynamic posture (elastic-driven strategy), whereas those envisioning the wheel naturally prolong contact duration, moderate stiffness, and smooth their force application over time (muscle-driven strategy). These outcome‐focused cues bypass technical instruction (e.g., “increase leg stiffness” or “shave 20 ms off contact time”) to accelerate self‐organization of movement patterns

Motor-learning research consistently demonstrates that externally oriented imagery accelerates self-organization of movement [123–125]. A simple yet powerful imagery guides this concept—the bouncy spring for short elastic actions, and the rolling wheel for long enduring efforts (Fig. 2). These two simple metaphors give athletes an outcome-focused cue (bounce vs. roll) that automatically shapes leg stiffness, contact time, and posture without overwhelming them with technical detail (e.g., “increase leg stiffness” or “reduce ground contact by 20 ms”). This mental representation becomes the bridge between the athlete’s identity and the demands of their objective. Recent research suggests a runner’s mental approach can affect their mechanics—for example, runners who focus on abstract holistic cues tend to adopt a more dynamic, elastic form [126]. Imaging studies confirm that rehearsing these metaphors activates the same sensorimotor networks used in the actual skill, strengthening the neural pathways required for efficient running and favoring relaxed yet economical mechanics [127].

Mental imagery is a powerful tool for optimizing running mechanics, enabling runners to internalize and adapt to new movement patterns with greater ease. By visualizing specific running styles, athletes can create mental imagery that guides their biomechanical adjustments and facilitates a smoother transition to an improved running form. These imagery strategies can be further categorized using a four-quadrant framework that distinguishes between broad versus narrow and internal versus external attentional focus—offering a structured way to align common coaching cues with the athlete’s cognitive orientation (see Tables 3, 4 for bouncy spring and rolling wheel imagery, respectively).

**Table 3:** The four-quadrant attentional focus framework associated with the bouncy spring imagery, commonly used to reinforce short elastic actions

Cues	Broad	Narrow
Internal	My body is light I am tall and elevated I am relaxed in the air	My feet rebound like carbon blades My tendons stretch and snap back like elastic bands My back is supple and toned
External	I fly I am light as air Like music, the rhythm lifts me	I am like an eagle Like a spring, I bounce effortlessly The ground is a trampoline The ground burns beneath me

The table maps internal and external cues according to broad and narrow attentional scope, offering guidance for optimizing focus during sustained running

**Table 4:** The four-quadrant attentional focus framework associated with the rolling wheel imagery, commonly used to reinforce long enduring efforts

Cues	Broad	Narrow
Internal	Energy flows through my body I am stable, strong, anchored	My feet push all the way through the toes—a long, driving push Strength in my thighs My breathing is powerful and effortless
External	The ground moves away behind me I’m running smoothly I feel the force of nature surrounding me Step by step, I move forward	I go where the water flows Deep into the ground I skim the surface I touch—I transition—I push I am like a wheel rolling forward

The table maps internal and external cues according to broad and narrow attentional scope, offering guidance for optimizing focus during sustained running

The mental imagery used depends on where a runner’s current pattern falls along the continuum of running styles. The bouncy spring analogy is ideal for short elastic running and refers to the spring-mass model of running (elastic-driven strategy). In the rolling wheel imagery, the body is imagined as a wheel rolling smoothly over the ground, with continuous ground contact that minimizes vertical oscillation and conserves energy (muscle-driven strategy). Picturing the legs moving in a circular wheel-like pattern can promote relaxation, forward momentum, and a steady rhythm, which aligns with a lower-impact grounded running style [96, 99]. The wheel image is intentionally idealized: it is not a literal model of running (not directly applicable to running) but a cognitive scaffold. It should be viewed as a simplified concept, and its limitations in reflecting the complexity of running biomechanics must be acknowledged. These extreme cases along the continuum help tailor imagery strategies to match the runner’s specific needs, performance goals, and running conditions.

This approach is grounded in a triptych of decision making:

- Sensation—the runner’s proprioceptive feelings and self-optimized spontaneous pattern (as profiled by the PIMP method).
- Observation
- the coach’s external view of kinematics, kinetics, and fatigue (external feedback and analysis).
- Reason—the rationale that links what is felt and seen to why a given cue or drill is prescribed.

These three pillars ensure that interventions are not only felt and seen but also understood. Within this framework, reasoning takes shape not only through language but also through motor imagery—where understanding is translated into intention. In movement, this intention becomes embodied through internal representations that guide motor execution, as supported by recent research in neuroimaging and motor learning [128, 129]. The brain responds favorably when tasks are anchored in existing motor patterns and intrinsic qualities, rather than externally imposed models. According to motor-learning theories, particularly those centered on self-referenced progression, optimal adaptation arises when training is based on one’s strengths—not on imitating unattainable ideals [130, 131].

This echoes the foundation laid by the PIMP approach: respect the spontaneous pattern, adapt it purposefully, and select cues—spring or wheel—that resonate with the athlete’s goals and context. In this way, performance becomes relative, self-driven, and sustainable. Mind to move is a practical methodology, ensuring that mental imagery, mechanical execution, and performance objectives remain in harmonious alignment.

## Conclusion

Good running technique is not a one-size-fits-all with a fixed standard but an emergent property of individualized adaptation. This review highlights that no universal running gait can be considered optimal; instead, biomechanical efficiency arises from the spontaneous self-organization of movement patterns that reflect each runner’s morphology, physiology, experience, objectives, and environmental context.

While the spring-mass model has greatly advanced our understanding—particularly of fast, elastic running—it shows clear limitations when applied to endurance locomotion. A more comprehensive view recognizes the existence of a continuum of running strategies, from elastic-driven to muscle-driven, where elastic and muscular contributions shift with speed, fatigue, and terrain. This reinforces the need for dynamic, person-environment interaction that respect variability rather than suppress it.

In applied contexts, the PIMP method provides a structured process for evaluating and refining running patterns, based on individualized observations rather than generalized ideals. Complementing this is the mind to move principle, which posits that movement optimization begins with internal representation. By using targeted mental imagery—such as the bouncy spring for short elastic actions or the rolling wheel for long enduring efforts—athletes align their intentions with their biomechanics, promoting efficient and intuitive adjustments.

Together, these approaches foster biomechanical coherence, the alignment of internal capacities with external demands. Sustainable performance and injury prevention do not emerge from imitation (mimicking elite form) but from cultivating techniques that resonate with the runner’s own physical and mental identity. Embracing this integrative, neuro-mechanical philosophy ensures that running becomes not only more effective but also more meaningful.

## Supplementary Material

Below is the link to the electronic supplementary material.

Supplementary file1 (DOCX 2867 kb)
