# Supplementary material for: Mind to Move: A Narrative Review of Individualized Running Biomechanics Beyond the Spring-Mass Model
Source: Sports Med. 2026 May 12;56(9):2121–38. doi: 10.1007/s40279-026-02449-w (PMC13687112; doi:10.1007/s40279-026-02449-w)
Supplement: Supplementary file 1 [file 40279_2026_2449_MOESM1_ESM.docx]

**Supplementary Information for**

**Mind to Move: A Narrative Review of Individualized Running Biomechanics Beyond the Spring-Mass Mode**l

Cyrille Gindre^1,2,3,4¶^, Thibault Lussiana^1,2,3,4¶^, Bastiaan Breine^1,5¶^, and Aurélien Patoz^1,6^*^¶^

^1^ Research and Development Department, Volodalen, Crissier 1023, Switzerland

^2^ Research and Development Department, Volodalen, Chavéria 39270, France

^3^ Université Marie et Louis Pasteur, SINERGIES, Besançon 25000, France

^4^ Université Marie et Louis Pasteur, Plateforme Exercice Performance Santé Innovation, Besançon 25000, France

^5^ Department of Movement and Sports Sciences, Ghent University, Ghent 9000, Belgium

^5^ Institute of Sport Sciences, University of Lausanne, Lausanne 1015, Switzerland

^¶^ These authors contributed equally to this work.

This supplementary section provides a detailed mechanical description of the spring-mass model introduced in the main manuscript. Whereas the main text presents the conceptual elements necessary to understand its relevance to running biomechanics, the complete mathematical formulation and key modeling assumptions are provided here to enhance transparency without interrupting the narrative flow. The supplement is organized to present (i) the formal derivation, parameter definitions, and governing equations of the model, (ii) its conceptual scope in relation to running mechanics, (iii) an illustrative worked example comparing model behavior with experimental data, and (iv) the main limitations of the framework and the implications of its simplifying assumptions. Readers seeking technical detail and model-specific nuance are referred to this Electronic Supplementary Information.

*S1. Mathematical formulation and assumptions*

The spring-mass model is a fundamental conceptual framework used to describe the dynamics of bouncing gaits, such as running [1, 2]. It consists of a point-mass representing the center of mass (COM), supported by a massless and linear spring that embodies the combined effect of all limbs in contact with the ground (Fig. S1). Using a linearly elastic spring implies that the ground reaction force is directly proportional to the leg’s shortening due to lower-limb joint flexion, an assumption that has proven to be a reasonable approximation [3]. The model exhibits alternating stance and aerial phases, and despite its extreme simplicity, it captures key features of running biomechanics, including ground reaction forces, energy fluctuations, and elastic energy storage.

**Figure S1.** Center of mass trajectory of human running, alongside the spring-mass model at key running events: foot-strike, mid-stance, toe-off, mid-flight, and contralateral foot-strike. The figure also illustrates the definition of contact and flight phases.

During the stance phase, the spring compresses as the point mass descends, storing elastic energy. As the spring recoils, this energy is released, propelling the mass upward and forward into the aerial phase, during which it follows a ballistic trajectory. This cyclical motion is governed by the following equations of motion (Newton’s second law) in the horizontal ($y$) and vertical ($z$) directions for the contact phase:

$$\ddot{y}(t)=\frac{kl_{0}}{m}\left[ 1-\frac{l(t)}{l_{0}} \right]\sin\theta(t)$$

$$\ddot{z}\left( t \right)=\frac{kl_{0}}{m}\left[ 1-\frac{l\left( t \right)}{l_{0}} \right]\cos\theta\left( t \right)-g,$$

and flight phase:

$$\ddot{y}(t)=0$$

$$\ddot{z}\left( t \right)=-g,$$

with initial conditions:

$$y\left( t=0 \right)=0$$

$$z\left( t=0 \right)=z_{0}$$

$$\dot{y}\left( t=0 \right)=\dot{y}_{0}$$

$$\dot{z}\left( t=0 \right)=0$$

$$\theta_{0.}$$

The model requires five initial (independent) parameters among the mass ($m$), the natural length of the spring ($l_{0}$), the initial vertical position of the point mass ($z_{0}$), the initial forward velocity of the point mass ($\dot{y}_{0}$), the leg angle at foot-strike ($\theta_{0}$), and the stiffness of the massless spring ($k$). In the above equations, $l(t)$ and $\theta\left( t \right)$ are the instantaneous leg length and leg angle, respectively. Stability is typically determined by fixing $m$, $l_{0}$, $z_{0}$, and $\dot{y}_{0}$ while solving for conditions where $\theta_{0}$ and $k$ yield a stable running cycle [4]. A fourth-order Runge-Kutta numerical scheme is commonly employed to integrate the system dynamics [2].

*S2. Scope – What the model captures*

The spring-mass model has been widely used as a reduced-order template to describe running mechanics at the system level [5-12]. Rather than reproducing the full physiological complexity of locomotion, it distills the runner into a point mass supported by a compliant leg, thereby providing an interpretable framework for examining dominant stance-phase dynamics. In particular, the model captures how the interaction between leg stiffness, contact time, and touchdown geometry can generate bouncing locomotion, in which mechanical energy is temporarily stored and subsequently returned through leg compression and recoil.

Conceptually, the value of the spring-mass model lies in its ability to explain how elastic-driven mechanics can emerge at the level of whole-body motion. When running behavior is characterized by relatively short ground contact times and spring-like leg behavior, this framework offers a compact mechanical description of how vertical support and forward progression can be produced through coordinated leg compression and recoil. In this context, it offers an interpretable mechanical reference for high-speed, rebound-like running patterns. By representing effective elastic energy storage and return during stance, the spring-mass framework helps explain how a substantial portion of mechanical energy can be recycled, thereby reducing the amount of net work that must be generated actively by muscle.

Importantly, the spring-mass model is not intended to represent neuromuscular control or muscle–tendon unit function in detail. It does not explicitly model muscle activation dynamics, multi-joint coordination, or energy dissipation processes. Instead, it should be interpreted as a mechanical template that is most informative when the dominant features of running can be approximated by conservative spring-like behavior. Under these conditions, it provides a clear link between external observables (e.g., COM motion and ground reaction forces) and simplified internal mechanics (e.g., an effective leg stiffness).

Although the traditional spring-mass model primarily describes bipedal running and quadrupedal trotting, modifications incorporating multiple leg springs can account for a broader range of gaits, including galloping. This versatility makes the model one of the most simple, comprehensive, and widely applicable frameworks for understanding running biomechanics [13, 14].

## *S3. A worked example of model–data agreement and deviation*

Figure S2 provides an illustrative comparison between experimental running data (black) and spring-mass model outputs (gray) across one step for two runners with the same level of expertise. The comparison includes the vertical COM trajectory, the vertical ground reaction force profile, and the “stiffness” curve (vertical force as a function of leg compression/decompression, ∆*L*).

In Panel A **(spring-mass runner)**, the model closely reproduces the measured COM trajectory and the timing and shape of the vertical force signal during stance. The force–compression relationship also shows a spring-like organization, indicating that changes in leg compression provide a meaningful proxy for vertical loading throughout stance. Mechanically, this suggests that the dominant step dynamics are consistent with a conservative spring-like interaction between the body and the ground. This panel therefore illustrates the conditions under which the spring-mass model functions effectively as an elastic-driven template of running mechanics.

**Figure S2.** Comparison of human (black) and model (gray) data for (A) a spring-mass runner and (B) a non-spring-mass runner. The comparison depicts the vertical center of mass (COM) trajectory and vertical ground reaction force as a function of a running step, with foot-strike (FS) and toe-off (TO) events represented. The figure also includes the “stiffness” curve, i.e., vertical force as a function of leg compression/decompression (∆*L*). Force is expressed in body weight (BW). Runners have the same level of expertise. **Panel B illustrates a stance pattern that deviates from spring-like symmetry and a conservative force–compression relationship.**

In contrast, panel B **(non-spring-mass runner)** shows systematic discrepancies between model predictions and experimental observations. Deviations are apparent in the COM trajectory and vertical force profile, and the force–compression relationship is less consistent with a conservative spring. A plausible mechanical interpretation is that stance behavior in this example is shaped by additional processes beyond passive elastic storage and return. Two broad contributors are especially relevant: (i) **greater energy dissipation**, occurring at touchdown and throughout stance (e.g., soft-tissue deformation, collision-related losses, tendon hysteresis), and (ii) **greater reliance on active muscle work**—including both positive and negative work—to regulate support and propulsion. Because these processes are not explicitly represented in the classic spring-mass formulation, the model cannot reproduce the observed dynamics when dissipation and/or actuation contribute substantially to the step mechanics.

Taken together, Figure S2 illustrates that the spring-mass model can provide a close approximation of running mechanics in elastic-driven conditions, yet may fail to capture patterns in which damping-like behavior and/or active work contributions play a larger role. This motivates extensions of spring-mass templates that incorporate active actuation and/or passive dissipation, as discussed in the main manuscript.

*S4. Limitations – What the model omits*

A central assumption of the classic spring-mass model is that running at constant speed can be represented by a conservative spring-like interaction during stance. This implies a simplified organization of stance dynamics and, in particular, a tendency toward symmetry between braking and propulsive phases within ground contact (equal braking and propulsive impulses). Empirical running data, however, frequently show braking–propulsion asymmetries, reflecting coordination strategies and mechanical constraints that are not represented by a purely conservative system [15, 16].

The model also cannot reproduce several well-documented features of ground reaction force profiles in human running. For example, vertical ground reaction forces often exhibit an initial impact transient followed by a later peak closer to mid-stance [17-19]. The early transient is commonly attributed to rapid deceleration of the foot and lower leg at touchdown and to collision-related effects. Such features are not predicted by the classic spring-mass model, which assumes a smooth and continuous spring-like loading response. In addition, the model typically assumes similar COM height at touchdown and takeoff, whereas in practice the COM is often higher at takeoff [15, 16, 20].

Another limitation concerns the representation of force application during stance. The spring-mass model neglects the forward translation of the point of force application under the foot. As a result, for a given contact length, it can overestimate horizontal ground reaction forces and mechanical work because the leg must be represented with a steeper touchdown angle relative to the vertical, increasing the horizontal-to-vertical force ratio [21].

Finally, the model is limited with respect to energetics. Because it does not include explicit muscle actuation, it cannot predict metabolic energy expenditure [22], which is central to gait selection and speed regulation [23, 24]. This omission also constrains its applicability to non-steady conditions such as acceleration, deceleration, and sloped running, where muscle work (concentric, eccentric, or isometric) becomes essential. Even in steady-level running, metabolic cost arises from energy dissipation within biological tissues and from active muscle work required to manage losses and regulate force—factors not represented in a purely conservative spring [25-29]. Therefore, spring-like running should be interpreted as **pseudo-elastic** rather than purely elastic. Overall, while the spring-mass model provides valuable system-level insight, its simplifying assumptions limit its ability to capture the full diversity of running patterns and energetics observed in humans.

*S5. References*

1.	McMahon TA, Cheng GC. The mechanics of running: how does stiffness couple with speed? J Biomech. 1990 1990/01/01/;23:65-78.

2.	Blickhan R. The spring-mass model for running and hopping. J Biomech. 1989 1989/01/01/;22(11):1217-27.

3.	Farley CT, Blickhan R, Saito J, Taylor CR. Hopping frequency in humans: a test of how springs set stride frequency in bouncing gaits. J Appl Physiol (1985). 1991 Dec;71(6):2127-32.

4.	Seyfarth A, Geyer H, Günther M, Blickhan R. A movement criterion for running. J Biomech. 2002 May;35(5):649-55.

5.	Blickhan R, Full RJ. Similarity in multilegged locomotion: Bouncing like a monopode. Journal of Comparative Physiology A. 1993 1993/11/01;173(5):509-17.

6.	Farley CT, Glasheen J, McMahon TA. Running springs: speed and animal size. J Exp Biol. 1993 Dec;185:71-86.

7.	Farley CT, González O. Leg stiffness and stride frequency in human running. J Biomech. 1996 Feb;29(2):181-6.

8.	Seyfarth A, Friedrichs A, Wank V, Blickhan R. Dynamics of the long jump. J Biomech. 1999 Dec;32(12):1259-67.

9.	Arampatzis A, Brüggemann GP, Metzler V. The effect of speed on leg stiffness and joint kinetics in human running. J Biomech. 1999 Dec;32(12):1349-53.

10.	Bullimore SR, Burn JF. Ability of the planar spring-mass model to predict mechanical parameters in running humans. J Theor Biol. 2007 Oct 21;248(4):686-95.

11.	Robilliard JJ, Wilson AM. Prediction of kinetics and kinematics of running animals using an analytical approximation to the planar spring-mass system. J Exp Biol. 2005 Dec;208(Pt 23):4377-89.

12.	Lipfert SW, Günther M, Renjewski D, Grimmer S, Seyfarth A. A model-experiment comparison of system dynamics for human walking and running. J Theor Biol. 2012 Jan 7;292:11-7.

13.	Gan Z, Wiestner T, Weishaupt MA, Waldern NM, David Remy C. Passive Dynamics Explain Quadrupedal Walking, Trotting, and Tölting. J Comput Nonlinear Dyn. 2016 Mar;11(2):0210081-2100812.

14.	Gan Z, Yesilevskiy Y, Zaytsev P, Remy CD. All common bipedal gaits emerge from a single passive model. J R Soc Interface. 2018 Sep 26;15(146).

15.	Cavagna GA. The landing–take-off asymmetry in human running. J Exp Biol. 2006;209(20):4051.

16.	Cavagna GA, Legramandi MA, Peyré-Tartaruga LA. The landing–take-off asymmetry of human running is enhanced in old age. J Exp Biol. 2008;211(10):1571.

17.	Kuitunen S, Komi PV, Kyröläinen H. Knee and ankle joint stiffness in sprint running. Med Sci Sports Exerc. 2002 Jan;34(1):166-73.

18.	Weyand PG, Bundle MW, McGowan CP, Grabowski A, Brown MB, Kram R, et al. The fastest runner on artificial legs: different limbs, similar function? J Appl Physiol (1985). 2009 Sep;107(3):903-11.

19.	Weyand PG, Sandell RF, Prime DN, Bundle MW. The biological limits to running speed are imposed from the ground up. J Appl Physiol (1985). 2010 Apr;108(4):950-61.

20.	Dewolf AH, Willems PA. A collision-based analysis of the landing-takeoff asymmetry during running. Computer Methods in Biomechanics and Biomedical Engineering. 2017 2017/10/30;20(sup1):S65-S6.

21.	Bullimore SR, Burn JF. Consequences of forward translation of the point of force application for the mechanics of running. J Theor Biol. 2006 2006/01/07/;238(1):211-9.

22.	Taylor CR, Heglund NC, Maloiy GM. Energetics and mechanics of terrestrial locomotion. I. Metabolic energy consumption as a function of speed and body size in birds and mammals. J Exp Biol. 1982 Apr;97:1-21.

23.	Long LL, 3rd, Srinivasan M. Walking, running, and resting under time, distance, and average speed constraints: optimality of walk-run-rest mixtures. J R Soc Interface. 2013 Apr 6;10(81):20120980.

24.	Srinivasan M, Ruina A. Computer optimization of a minimal biped model discovers walking and running. Nature. 2006 2006/01/01;439(7072):72-5.

25.	Lai A, Lichtwark GA, Schache AG, Lin YC, Brown NA, Pandy MG. In vivo behavior of the human soleus muscle with increasing walking and running speeds. J Appl Physiol (1985). 2015 May 15;118(10):1266-75.

26.	Lichtwark GA, Bougoulias K, Wilson AM. Muscle fascicle and series elastic element length changes along the length of the human gastrocnemius during walking and running. J Biomech. 2007;40(1):157-64.

27.	Ker RF. The time-dependent mechanical properties of the human heel pad in the context of locomotion. J Exp Biol. 1996 Jul;199(Pt 7):1501-8.

28.	Lichtwark GA, Wilson AM. In vivo mechanical properties of the human Achilles tendon during one-legged hopping. J Exp Biol. 2005 Dec;208(Pt 24):4715-25.

29.	Riddick RC, Kuo AD. Soft tissues store and return mechanical energy in human running. J Biomech. 2016 Feb 8;49(3):436-41.
